# Behavioural withdrawal during an acute stress test as a marker of psychobiological vulnerability in hereditary angioedema

**DOI:** 10.3389/fimmu.2026.1784326

**Published:** 2026-03-03

**Authors:** Luca Ranucci, Francesca Perego, Aida Zulueta, Clara Gino, Azzurra Cesoni Marcelli, Lorenza Chiara Zingale, Laura Adelaide Dalla Vecchia, Beatrice De Maria, Alessandra Gorini

**Affiliations:** 1 Istituti Clinici Scientifici Maugeri IRCCS, PsyCaRe Lab – Laboratorio di Psicologia per un Approccio Integrato alle Patologie Cardiopolmonari e le Malattie Rare in Ambito Cardiovascolare, Milan, Italy; 2 Department of Internal Medicine and Rehabilitation, Istituti Clinici Scientifici Maugeri IRCCS, Milan, Italy; 3 Istituti Clinici Scientifici Maugeri IRCCS, Laboratorio di Ricerca sui Biomarcatori Neurologici (LaBioN), Milan, Italy; 4 Bioengineering Laboratory, Istituti Clinici Scientifici Maugeri IRCCS, Milan, Italy; 5 Department of Cardiology, Istituti Clinici Scientifici Maugeri IRCCS, Milan, Italy; 6 Dipartimento di Scienze Cliniche e di Comunità, Dipartimento di Eccellenza 2023-2027, Università degli Studi di Milano, Milan, Italy

**Keywords:** hereditary angioedema, inflammation, phenotype, preventive strategies, psychological assessment, rare disease, socially evaluated cold pressor test (SeCPT), stress reactivity

## Abstract

**Introduction:**

Hereditary angioedema due to C1-inhibitor deficiency (HAE-C1INH) features clinical heterogeneity and stress-triggered attacks. Behavioral tolerance to acute stress may reveal vulnerability profiles beyond standard clinical descriptors. This study aimed to characterize stress response patterns and compare groups based on behavioral tolerance.

**Methods:**

HAE-C1INH patients underwent the Socially Evaluated Cold Pressor Test (SECPT) and were stratified as Completers or Non-completers (early withdrawal). Stress appraisal, cardiovascular parameters (heart rate, HR; systolic/diastolic arterial pressure, SAP/DAP), and plasma cytokines (IL-1β, TNF-α, IL-6) were assessed. Disease control and quality of life were measured via Angioedema Control Test (AECT) and Angioedema Quality of life (AE-QoL) questionnaires.

**Results:**

Twenty patients were enrolled (15 Completers and 5 Non-completers). Non-completers showed poorer disease control (10.6 ± 5.5 vs 14.5 ± 2.2; p ≤ 0.05) and worse AE-QoL, particularly in the Functioning (8.6 ± 4.3 vs 4.7 ± 1.7; p ≤ 0.05) and Fatigue/Mood (13.6 ± 7.1 vs 10.5 ± 3.5; p ≤ 0.05) domains. They reported higher stress (91 ± 8.9 vs 50.5 ± 33.7; p ≤ 0.05), pain (87.8 ± 12.8 vs 50.1 ± 31.3; p ≤ 0.05) and unpleasantness (83 ± 19.9 vs 49.5 ± 30.5; p ≤ 0.05) during the SECPT. Non-completers displayed an attenuated SAP response relative to Completers (128.3 ± 18.0 vs 148.9 ± 18.3 mmHg; p ≤ 0.05). Inflammatory profiles also diverged: Non-completers showed higher IL-6 levels at 40 minutes after SECPT (3.5 ± 1.1 vs 2.2 ± 0.7 pg/ml; p ≤ 0.05) and opposite TNF-α trajectories compared with Completers (0.9 ± 1.0 vs -0.5 ± 0.9 pg/ml; p ≤ 0.05).

**Conclusion:**

Early withdrawal during SECPT identifies a vulnerable HAE-C1INH subgroup with distinct psychological, cardiovascular, and inflammatory patterns.

## Introduction

Hereditary angioedema due to C1-Inhibitor deficiency (HAE-C1INH) is a rare, genetic condition characterized by unpredictable and potentially life-threatening swellings (1, 2). Such unpredictability leads to a substantial burden, manifesting as anxiety, depression, anticipatory worry, and reduced Quality of Life (QoL) (3–5), which often persists during attack-free periods (6). Although current therapeutic options, such as On-Demand (ODT) and Long-Term Prophylaxis (LTP) therapies, can effectively control clinical manifestations, attacks may still occur, and the disease burden frequently remains elevated (7).

This persistent burden is compounded by the remarkable and largely unexplained clinical heterogeneity of HAE-C1INH, with phenotypes ranging from asymptomatic to symptomatic with a high variability of the number of attacks that can be frequent and highly debilitating (8). The determinants of individual susceptibility to specific attack triggers are unclear. Consequently, there is a critical need to move beyond standard clinical descriptors and identify novel biomarkers capable of capturing this heterogeneity and stratifying patient vulnerability with the aim of implementing further preventive strategies.

Notably, patients frequently report psychobiological stress as an attack trigger (9), and increased attack rates have been documented during major population-level stressors (10–12). These observations point to a vicious cycle in which stress precipitates attacks, and these attacks in turn amplify stress and illness-related anxiety (9). Given this complexity, research should focus on a multidimensional framework, where biological, psychological, and behavioral domains are interpreted as co-occurrent phenomena that represent the patients’ experience (9).

To address this gap, through a multidimensional lens, we employed the Socially Evaluated Cold Pressor Test (SECPT), a validated acute stress paradigm that robustly activates both the sympathetic–adrenomedullary (SAM) and hypothalamic–pituitary–adrenal (HPA) systems (13). Crucially, beyond physiological indices, the SECPT provides a behavioral marker of stress endurance, defined as task completion versus early withdrawal. In healthy subjects, where approximately 15% of participants fail the task (14), early withdrawal has been linked to greater pain sensitivity, higher perceived stress, and blunted cardiovascular reactivity (CVR) (14, 15). Previous evidence also showed that, during SECPT, HAE-C1INH patients exhibit heightened perceived stress and elevated baseline inflammation compared to healthy controls despite having comparable CVR (16).

We reasoned that early withdrawal in HAE-C1INH patients, provides a behavioral index of stress intolerance that may capture inter-individual differences not detectable through standard clinical descriptors. Therefore, the aim of this study was to characterize and compare the stress reactivity patterns in HAE-C1INH patients associated with SECPT completion to identify potential markers of vulnerability.

## Materials and methods

### Participants

We considered the cohort of HAE-C1INH patients that were studied for the FRoSEn study (16). In this pilot study HAE-C1INH patients were consecutively enrolled during ambulatory visits from June 2023 to April 2024 at the IRCCS Istituti Clinici Scientifici Maugeri of Milan. Inclusion criteria were: (i) a documented diagnosis of HAE-C1INH and (ii) an age between 18 and 65 years. Exclusion criteria included: (i) presence of any other chronic disease requiring chronic treatment (e.g., hypertension, diabetes, autoimmune diseases), (ii) an active acute illness, (iii) SARS-CoV-2 infection in the previous 3 months (17), (iv) HAE attack occurring in the week preceding the experimental session and (v) HAE attack in the following 72 hours (a posteriori exclusion).

A formal sample size calculation was not applicable due to the lack of data for comparison in HAE-C1INH patients. All analyses were exploratory due to the pilot sample size.

The study was conducted in accordance with the Declaration of Helsinki and was approved by the Ethics Committee of the IRCCS Istituti Clinici Scientifici Maugeri (approval number 2774 CE; date of approval 31 May 2023) and was registered on ClinicalTrials.gov (NCT06414252). All participants provided written informed consent before their inclusion in the study.

### Study procedure

**Figure 1:**
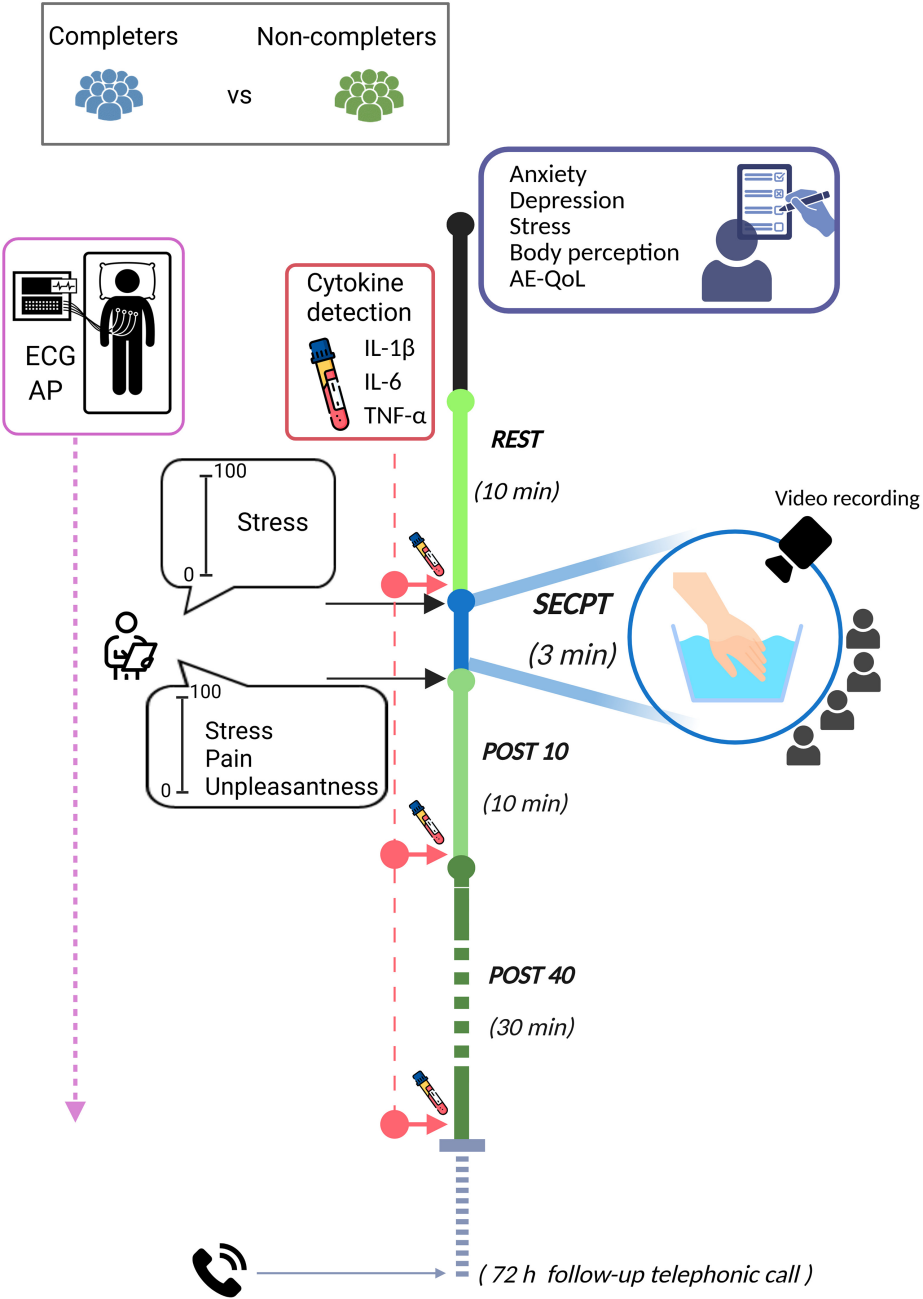
Schematic representation of the experimental protocol. The figure was created in Biorender.com.

An overview of the experimental protocol is provided in **Figure 1**, while a detailed description of the protocol is presented elsewhere (16). Briefly, experimental sessions were conducted in a quiet room with a comfortable temperature, between 9 a.m. and 12 p.m. to control for diurnal variations in biochemical parameters, and participants were instructed to avoid consuming caffeine prior to the experimental protocol. All participants underwent a medical visit to verify the inclusion and exclusion criteria and to collect demographic and clinical data. A venous catheter was subsequently inserted to allow for repeated blood sampling, avoiding multiple punctures. Participants were also instrumented for continuous monitoring of heart rate (HR) and non- invasive arterial blood pressure (AP).

In summary, the protocol consisted of the following phases (**Figure 1**):

REST (Baseline): a set of questionnaires assessing sociodemographic and psychological variables was administered, then a 10-minute resting period in a supine position in a quiet room started. A baseline blood sample was collected at the end of this period.

SECPT (Stress Task): participants, remaining in a supine position, were instructed to immerse their hand in cold water maintained at 4°C for a maximum of 3 minutes. They were informed that they could remove their hand at any time if the perception became intolerable. To induce social evaluation, participants were videotaped and instructed to look into the camera for the entire duration of the immersion, while an experimenter monitored them and took notes. Immediately before the hand immersion, participants rated how stressed they were on a Visual Analogue Scale (VAS) (0 = “not at all” to 100 = “very much”).

Subjective Ratings: immediately after removing their hand from the water, participants rated how unpleasant, stressful, and painful the experience had been on a VAS (0 = “not at all” to 100 = “very much”).

POST10 (Recovery 1): a 10-minute recovery period immediately started after the SECPT. A second blood sample was collected at the end of this phase.

POST40 (Recovery 2): an additional 30-minute recovery period (40 minutes post SECPT) ended with a third blood sample collection. Continuous physiological signals were monitored throughout all phases.

A follow-up phone call was made 72 hours after the session to verify the potential occurrence of HAE-C1INH attacks.

Following the experimental session, patients were categorized into two subgroups based on their SECPT performance. Patients who completed the 3-minute protocol were classified as “Completers”, while those who withdrew before the 3-minute cut-off were classified as “Non-completers”.

### Psychological scales

A battery of self-report questionnaires was administered to assess a range of psychological outcomes at baseline. The Hospital Anxiety and Depression Scale (HADS) (18) was administered to measure anxiety and depressive symptoms. Stress perception during the last month was measured with the Perceived Stress Scale (PSS) (19). Pain-related cognitions were investigated using the Pain Catastrophizing Scale (PCS) (20). Then, given the somatic nature of HAE-C1INH, participants’ relationship with their body was also explored through several scales. The Functionality Appreciation Scale (FAS) (21) and the Body Appreciation Scale (BAS) (22) were used to assess appreciation for the body’s capabilities and overall acceptance of one’s body, respectively. The Multidimensional Assessment of Interoceptive Awareness (MAIA) (23) was administered to evaluate multiple dimensions of interoception, such as the ability to notice, listen to, and trust bodily sensations. To assess personality traits relevant to perseverance, the Short Grit Scale (Grit-S) (24) was included; this 8-item questionnaire measures “grit,” defined as the tendency to apply passion and sustained effort toward long-term goals. Lastly, angioedema specific outcomes were quantified using the Angioedema Quality of Life (AE-QoL) (25) questionnaire, a 17-item scale, to measure disease-specific impairment across four domains (Functioning, Fatigue/Mood, Fears/Shame, and Food). It is scored as a percentage on a scale of 0 to 100, with lower scores indicating less impairment and better quality of life. Disease control was assessed by the 4-item Angioedema Control Test (AECT) (26), which provides a score from 0 to 16, with higher scores indicating better disease control.

### Physiological measures

As described elsewhere (16), continuous physiological signals were recorded, including a continuous electrocardiogram (ECG, LAB3 device, Marazza, Monza, Italy) and non-invasive AP via a photoplethysmography device (Finometer, Finapres Medical System) positioned on the middle finger of the right hand. ECG was sampled at 1000 Hz, while AP at 250 Hz. From these signals, beat-to-beat time series of RR intervals, systolic arterial pressure (SAP), and diastolic arterial pressure (DAP) were derived for subsequent analysis. RR was defined as the temporal distance between two consecutive R peaks detected on the ECG, while SAP and DAP as the maximum and minimum values of the AP signal inside each RR, respectively.

### Biochemical parameters analysis

Six milliliters of blood from each participant were drawn into EDTA-coated tubes at three time points: REST, POST10, and POST40. To obtain plasma, the blood samples were centrifuged for 15 minutes at 2,000 × g within 30 minutes of collection. The plasma fraction was then divided into 500 µL aliquots, quickly frozen and kept at −80 °C until further experiments. Plasma levels of Interleukin-1 beta (IL-1β), Interleukin-6 (IL-6) and Tumor Necrosis Factor-alpha (TNF-α) were measured using high-sensitive enzyme-linked immunosorbent assays (ELISA). The following commercial kits were used: IL-1β (E-HSEL-H0001, ElabScience, USA), IL-6 (950.035.096, Diaclone, France), and TNF-α (EIA-4641, DRG, Germany). Each assay included blanks, calibration standards, quality controls, and plasma samples, all tested in duplicate using 96-well microplates, in accordance with the manufacturers’ protocols. Optical density was measured at 450 nm using an automated ELISA reader. Cytokine concentrations were expressed in picograms per milliliter (pg/mL), and the mean value of the duplicates was used for statistical analysis.

### Data processing and statistical analysis

Statistical analyses were performed using Sigmaplot (v.14.0, Systat Software, San Jose, CA, USA). The Shapiro-Wilk test was applied to check the normality of the distribution. Unpaired Student t test, or Mann-Whitney rank sum test in case of not-normal distribution, was applied to compare baseline demographic, clinical, psychological characteristics, subjective ratings of stress, pain, and unpleasantness, psychophysiological profile, cardiovascular and inflammatory response between the Completer and Non-completer groups. Fisher’s exact test was used for categorical variables. A p<0.05 was considered as significant.

## Results

### Demographic and clinical characteristics

The study cohort comprised 20 adult patients with HAE-C1INH (mean age: 44.7 ± 14.8 years; 11 females). Of these, 15 (75%) managed to complete the SECPT while 5 (25%) withdrew their hand before the 3-minute cut-off. No attacks were reported within the next 72 hours. Demographic data and key clinical variables relevant to the disease (e.g., Attack rate, LTP) and its impact on daily life (e.g., AE-QoL, AECT) are summarized in **Table 1**. Most patients (18 out of 20) were on LTP regimen at the time of the study. Comparisons between Completers and Non-completers revealed significant differences in AECT and AE-QoL. Specifically, Completers reported better disease control and lower impairment in the “Functioning” and “Fatigue/Mood” domains of the AE-QoL. Conversely, no significant differences were observed regarding other baseline variables.

**Table 1:** Demographic and baseline clinical characteristics of the total sample and the two groups (Completers and Non-completers).

Variables	HAE Total (n=20)	Completers (n=15)	Non-completers (n=5)
Age, yrs	44.7 ± 14.8	45.0 ± 14.6	44.0 ± 17.1
Sex, n males/females	9/11	8/7	1/4
BMI, kg·m^-2^	25.0 ± 3.8	25.3 ± 3.3	23.9 ± 5.5
Education, yrs	14.0 ± 4.0	14.2 ± 3.8	13.4 ± 5.1
Age at diagnosis, yrs	18.6 ± 12.4	21.5 ± 12.7	10.0 ± 6.5
Years from diagnosis, yrs	26.3 ± 11.3	23.7 ± 9.9	34.2 ± 13.0
Attack rate in the last 6 months, n	2.9 ± 8.8	3.34 ± 10.2	1.6 ± 1.7
No LTP, n (%)	2.0 (10.0)	2.0 (13.3)	0 (0)
AE-QoL	29.3 ± 12.2	25.9 ± 6.2	39.6 ± 20.1
Functioning	5.7 ± 3.0	4.7 ± 1.7*	8.6 ± 4.3
Fatigue/Mood	9.6 ± 4.7	8.4 ± 3.4*	13.2 ± 6.8
Fears/Shame	11.3 ± 4.6	10.5 ± 3.5	13.6 ± 7.1
Food	2.7 ± 1.7	2.3 ± 0.6	4.2 ± 3.0
AECT	13.5 ± 3.5	14.5 ± 2.2*	10.6 ± 5.5
HADS_A	7.3 ± 4.5	7.2 ± 4.1	7.6 ± 6.1
HADS_D	3.1 ± 3.3	2.9 ± 3.4	3.8 ± 3.3
FAS	3.9 ± 0.7	4.0 ± 0.6	4.0 ± 0.8
BAS	37.7 ± 7.2	37.4 ± 7.4	38.6 ± 7.1
MAIA			
Not-Worrying	2.5 ± 1.1	2.4 ± 1.1	3.1 ± 1.0
Noticing	3.3 ± 1.1	3.2 ± 1.0	3.6 ± 1.2
Not-Distracting	2.2 ± 0.8	2.3 ± 0.8	2.1 ± 0.6
Attention Regulation	2.7 ± 1.1	2.7 ± 1.0	2.6 ± 1.3
Emotional Awareness	3.5 ± 1.1	3.4 ± 1.2	3.6 ± 1.0
Self-Regulation	2.7 ± 1.4	2.9 ± 1.4	2.1 ± 1.3
Body Listening	2.5 ± 1.3	2.6 ± 1.3	2.1 ± 1.5
Trusting	3.3 ± 1.1	3.4 ± 1.0	2.7 ± 1.4
PSS	17.1 ± 7.2	16.9 ± 7.2	18 ± 8.2
Grit-S	3.8 ± 0.5	3.7 ± 0.5	4.2 ± 0.6
PCS	19.7 ± 12.6	20.5 ± 13.7	17.4 ± 9.5
Helplessness	7.0 ± 6.1	7.0 ± 6.6	7.0 ± 4.8
Rumination	9.1 ± 4.8	9.9 ± 5.1	7.0 ± 3.6
Magnification	3.5 ± 2.5	3.6 ± 2.8	3.4 ± 1.5

BMI, Body Mass Index; HAE, Hereditary Angioedema due to C1INH deficiency; LTP, Long-Term Prophylaxis; AE-QoL, Angioedema Quality of Life Questionnaire; AECT, Angioedema Control Test. HADS, Hospital Anxiety and Depression Scale; FAS, Functionality Appreciation Scale; BAS, Body Appreciation Scale; MAIA, Multiphasic Assessment of Interoceptive Awareness; PSS, Perceived Stress Scale; PCS, Pain Catastrophizing Scale. Categorical variables are reported as absolute numbers (%), and continuous data are reported as mean ± standard deviation. Questionnaires results are reported as scores; *Completers vs Non-completers, *p ≤* 0.05.

### Psychological response

**Figure 2:**
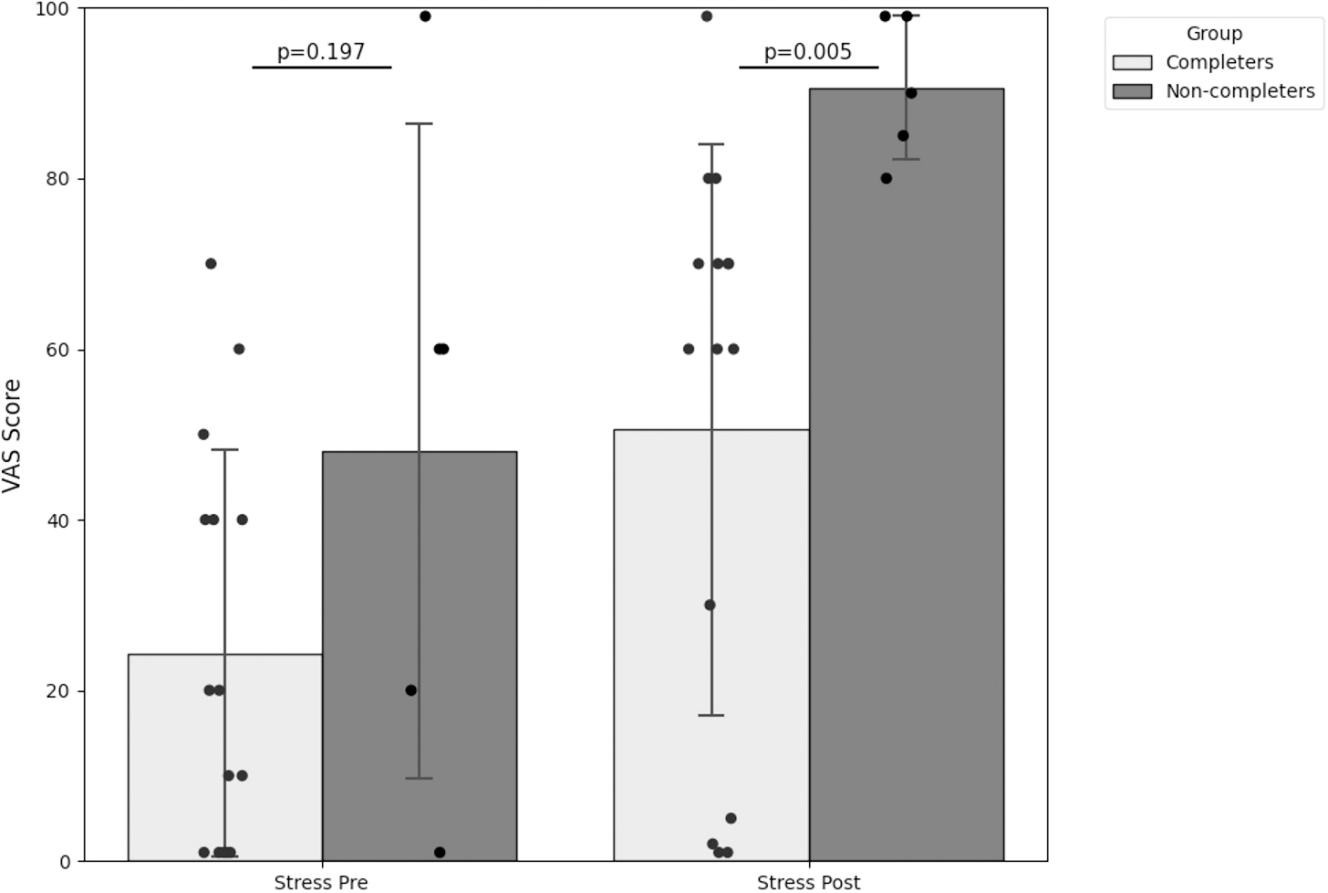
Group comparison of stress ratings (VAS stress) prior and immediately after the SECPT. Stress Pre, VAS stress before SECPT; Stress Post, VAS stress after SECPT.

**Figure 3:**
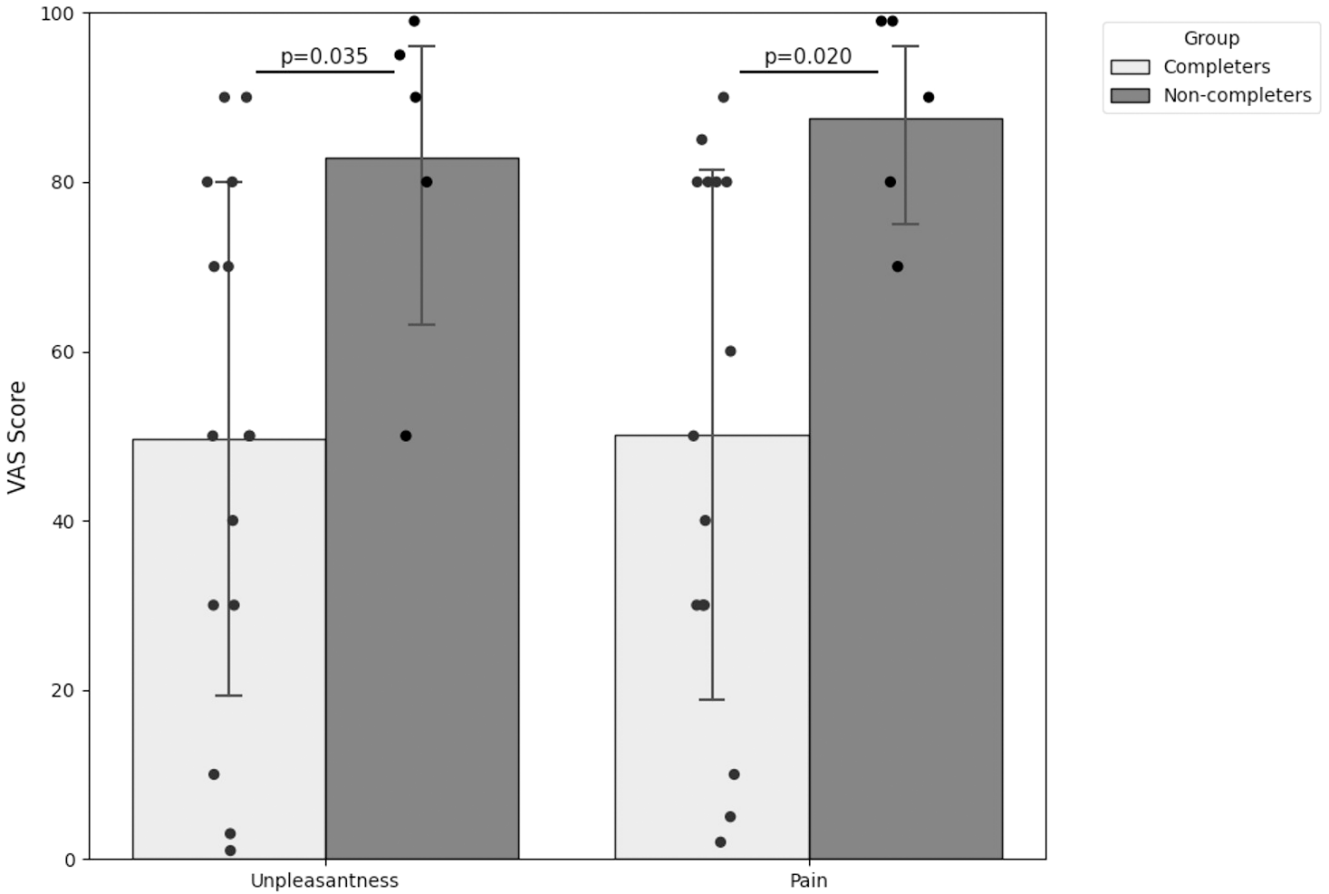
Group comparison of unpleasantness and pain ratings (VAS unpleasantness and VAS pain) immediately after SECPT. Unpleasantness, perceived unpleasantness during SECPT; Pain, perceived pain during SECPT.

**Figure 2** shows the comparison of stress appraisal prior and immediately after the SECPT phase, while **Figure 3** illustrates the comparison of perceived pain and unpleasantness immediately after SECPT. Group comparison revealed distinct profiles. Non-completers exhibited higher baseline stress levels compared to Completers (47.8 ± 38.6 vs. 24.0 ± 24.1; p=0.197) and reported a significantly higher, near-maximal surge in stress immediately after the stress task (91.0 ± 8.9 vs. 50.5 ± 33.7; p=0.05).

Furthermore, the subjective appraisal of the stimulus differed substantially: Non-completers experienced the task as significantly more painful (87.8 ± 12.8 vs. 50.1 ± 31.3; p=0.02) and unpleasant (83.0 ± 19.9 vs. 49.5 ± 30.5; p=0.03).

### Cardiovascular response

Cardiovascular reactivity data are presented in **Table 2**. Non-completers registered significantly lower SAP during SECPT compared to Completers. This difference was also present in the POST40 phase, where Non-completers maintained a lower SAP.

**Table 2:** Between group comparison of heart rate and arterial blood pressure response.

Experimental phases	Completers (N = 15)	Non-completers (N = 5)	p
REST			
HR, bpm	59.3 ± 8.6	67.0 ± 13.6	0.152
SAP, mmHg	122.8 ± 16.4	119.2 ± 12.9	0.666
DAP, mmHg	77.8 ± 11.1	76.4 ± 6.6	0.792
SECPT			
HR, bpm	66.5 ± 8.1	75.2 ± 12.7	0.090
SAP, mmHg	148.9 ± 18.3	128.3 ± 18.0	0.042*
DAP, mmHg	92.4 ± 12.7	82.9 ± 9.5	0.144
POST10			
HR, bpm	58.5 ± 6.3	65.1 ± 10.9	0.110
SAP, mmHg	122.7 ± 17.4	119.7 ± 13.1	0.729
DAP, mmHg	78.1 ± 11.3	79.4 ± 6.4	0.810
POST40			
HR, bpm	58.6 ± 7.1	64.3 ± 9.4	0.161
SAP, mmHg	129.8 ± 15.2	113.2 ± 5.2	0.049*
DAP, mmHg	82.4 ± 12.9	79.2 ± 8.7	0.618

REST, 10 minutes resting phase; SECPT, Socially Evaluated Cold Pressor Test; POST10, 10 minutes after SECPT; POST40, 30 minutes after POST10; HR, Heart Rate; SAP, Systolic Arterial Pressure; DAP, Diastolic Arterial Pressure. Data are reported as Mean ± Standard Deviation. *Completers vs Non-completers, *p ≤* 0.05.

When comparing group trajectories across experimental phases, Non-completers exhibited an attenuated AP response to the stressor compared to Completers (Appendix A). Specifically, the increase in SAP from REST to SECPT was significantly smaller among Non-completers (9.1 ± 7.9 vs 26.2 ± 12.0 mmHg; p<0.01). Similarly, the DAP response was significantly attenuated in Non-completers (6.4 ± 4.5 vs 14.5 ± 5.2 mmHg; p<0.01). From SECPT to POST10 phase, Non-completers demonstrated a significantly smaller DAP reduction compared to the recovery observed in Completers (-3.4 ± 4.0 vs -14.2 ± 7.9 mmHg; p<0.01).

### Inflammatory response

**Table 3** reports data on inflammatory cytokines. Between groups comparison showed trending differences regarding IL-6 levels at REST, and at POST10 in which Non-completers showed higher concentrations than Completers. This trend reached statistical significance during the POST40 phase, in which the Non-completers exhibited significantly higher levels of IL-6 (2.2 ± 0.7 vs 3.5 ± 1.1 pg/ml; p=0.014). Regarding IL-1β and TNF-α, the analysis did not yield statistically significant differences between the two subgroups at any specific time point.

**Table 3:** Plasma concentrations of pro-inflammatory cytokines (IL-1β, TNF-α, IL-6) across experimental phases (REST, POST10, POST40) of the two groups (Completers vs Non-completers).

Experimental phases	Completers (N = 15)	Non-completers (N = 5)	p
REST			
IL-1β, pg/ml	19.5 ± 29.5	25.4 ± 26.2	0.431
TNF-α, pg/ml	4.3 ± 1.1	3.7 ± 2.0	0.394
IL-6, pg/ml	2.3 ± 1.1	3.4 ± 1.2	0.090
POST10			
IL-1β, pg/ml	19.4 ± 29.3	23.9 ± 21.9	0.379
TNF-α, pg/ml	4.1 ± 0.9	3.9 ± 2.4	0.643
IL-6, pg/ml	2.5 ± 1.1	3.5 ± 1.2	0.099
POST40			
IL-1β, pg/ml	19.6 ± 29.5	24.0 ± 23.9	0.379
TNF-α, pg/ml	3.8 ± 0.9	4.6 ± 2.0	0.926
IL-6, pg/ml	2.2 ± 0.7	3.5 ± 1.1	0.014*

REST,10 minutes resting phase; POST10, 10 minutes after Socially Evaluated Cold Pressor Test; POST40, 30 minutes after POST10; IL-1β, Interleukin-1 beta; TNF-α, Tumor Necrosis Factor-alpha; IL-6, Interleukin-6. Data are presented as Mean ± Standard Deviation. *Completers vs Non-completers, *p ≤* 0.05.

However, with respect to TNF-α trajectories across the experimental phases, the two groups showed opposite dynamics (Appendix B). Between REST and POST40, Completers showed a decreasing trend while Non-completers exhibited an increasing one (-0.46 ± 0.86 vs. 0.93 ± 0.97 pg/ml; p=0.008) and this difference was also present between POST10 and POST40 phases (-0.26 ± 0.76 vs 0.72 ± 0.75 pg/ml; p=0.024).

## Discussion

This pilot study investigated whether behavioral withdrawal during a standardized acute stress task (SECPT non-completion) captures clinically relevant heterogeneity in patients affected by HAE-C1INH. Clinical heterogeneity and persistent burden are widely recognized in HAE-C1INH, including impaired quality of life and disease control despite available therapies (5, 7, 25). In parallel, patients frequently report stress as a trigger, and increased attack rates have been described during major population-level stressors (10, 12). Within this context, we observed that early SECPT withdrawal was associated with higher disease burden, greater subjective distress during the stressor, an attenuated arterial blood pressure response, and distinct inflammatory trajectories during the experimental phases. Together, these converging signals suggest that SECPT performance may offer a pragmatic behavioral index of stress tolerance that complements standard clinical descriptors.

### Behavioral withdrawal as a marker of vulnerability

The SECPT is a well-validated laboratory paradigm that robustly activates stress-related systems while providing an objective behavioral endpoint (task completion vs early withdrawal) (13, 14). In our sample, non-completion was not explained solely by greater unpleasantness; rather, Non-completers combined high subjective distress (stress, pain, unpleasantness) with poorer disease control and QoL impairment. Although causality cannot be inferred, the alignment between behavioral avoidance and clinically meaningful burden supports the interpretation of early withdrawal as a candidate marker of vulnerability within HAE-C1INH.

### AP response patterns during acute stress

Non-completers showed an attenuated increase in SAP/DAP during the stress phase and a less pronounced recovery pattern relative to Completers. Rather than using the broader label of “blunted reactivity”, our data specifically indicate an attenuated AP response in this experimental context. Reduced cardiovascular reactivity has been linked to altered stress regulation and adverse behavioral/health correlates in other settings (27–29). In HAE-C1INH, where unpredictability and anticipatory worry are common (7, 25), limited pressor mobilization and/or inefficient recovery may contribute to perceived uncontrollability and avoidance behavior. This finding adds granularity to our previous observation that HAE-C1INH exhibit heightened perceived stress despite displaying a cardiovascular reactivity comparable to that of healthy subjects (16).

Another possible interpretation of our results is that stress-induced release of bradykinin, a potent endothelial vasodilator, may functionally oppose the sympathetic vasoconstriction triggered by the SECPT (30). In this scenario, the elevated HR in Non-completers may represent a compensatory but ineffective chronotropic effort, attempting to counteract the bradykinin-mediated vasodilation (31, 32). Notably, this pattern of heightened sympathetic drive failing to stabilize vascular tone mirrors the autonomic dysregulation often hypothesized to occur in the prodromal phase of attacks (33), reinforcing the concept that this hemodynamic inefficiency may mirror the physiological instability that predisposes patients to attacks.

Given the small subgroup size, these physiological findings remain exploratory and should be replicated with more complex autonomic measures in stable conditions (i.e. REST and recovery phases) by HR and AP variability analysis and baroreflex sensitivity assessment.

### Inflammatory trajectories

Beyond subjective appraisal and pressor indices, Non-completers displayed distinct inflammatory dynamics, including higher IL-6 during late experimental phase and divergent TNF-α trajectories relative to Completers. Similar findings were found in a recent study on pro-inflammatory cytokine concentrations in the overall HAE-C1INH population. Participants showed significantly higher cytokine concentrations compared to healthy controls (34). Stress-related immune changes are shaped by bidirectional neuro-immune pathways and regulatory loops (35, 36). In HAE-C1INH, inflammatory signaling is also embedded within disease-specific mechanisms such as the contact system and bradykinin-related pathways (30, 32). Accordingly, our cytokine findings should be interpreted as preliminary evidence of differential immune response associated with behavioral stress intolerance, rather than as definitive mechanistic proof.

### Integrative interpretation and implications

Taken together, our findings converge on the hypothesis that SECPT non-completion identifies a subgroup with (i) greater subjective threat/pain appraisal, (ii) less efficient AP mobilization, and (iii) a more sustained inflammatory response profile. Predictive processing accounts, including the Embodied Predictive Interoceptive Coding (EPIC) framework (37), offer one plausible interpretative lens: illness burden may act as a negative prior that biases the organism toward conservative, energy-preserving responses when incoming signals are appraised as costly or threatening. In parallel, models emphasizing sickness behavior and effort-based decision-making highlight how inflammatory and interoceptive signals can shift motivation toward withdrawal (38), consistent with energy-conserving strategies described in other physiological domains (39). The observed attenuated AP response and the concomitant inflammatory patterns match the “Type 4” profile of allostatic load described by McEwen (36), defined as an “Inadequate Response”, where the failure of one system to mount a sufficient response leads to the compensatory hyperactivity of another. On one hand, the inadequate cardiovascular engagement may lead to a failure of the vagal anti-inflammatory reflex (35). On the other hand, in line with the EPIC model, inaccurate predictions can lead to a pro-inflammatory state, in our context the specific increase in TNF-α suggests the activation of a stress-reactive kinin-mast cell axis (30). Specifically, we hypothesize that in these vulnerable individuals, acute stress may trigger a low-grade release of bradykinin. This mediator could simultaneously account for our findings by blunting the pressor response through vasodilation and promoting a pro-inflammatory state via mast cell activation, resulting in the observed TNF-α surge. This finding is particularly significant as it may represent the mechanistic bridge between acute stress and attack vulnerability. While TNF-α is a known marker of systemic inflammation, its sustained rise in Non-completers suggests a failure to buffer the stress-induced activation of the contact system. Biologically, the released TNF-α can further sensitize the endothelium and promote mast cell degranulation (40), potentially lowering the threshold for bradykinin-mediated vascular leakage (41). Consequently, a feed-forward loop is established: it is biologically plausible that bradykinin signaling not only blunts the pressor response but also promotes downstream cytokine release from sensitized mast cells or endothelium (30). This may create a self-sustaining vicious cycle where the inability to mount a hemodynamic response translates directly into unbuffered inflammatory signaling.

These frameworks should be presented as hypotheses that generate testable predictions. Clinically, if replicated, behavioral withdrawal during standardized stress exposure could support stratification of patients who may benefit from targeted stress regulation and coping interventions integrated with routine care.

### Limitations

This study has several limitations. First, it is a pilot investigation with a small sample, the Non-completer group was particularly small, and most of them were on LTP. This limits statistical power, stability of subgroup estimates and the interpretation of the contribution of prophylaxis on the results. Second, multiple outcomes were examined; therefore, results should be interpreted as exploratory, with emphasis on patterns and effect sizes rather than definitive inference. Third, we did not include neuroendocrine measures (e.g., cortisol). This decision was deliberately taken to avoid the confounding effects of its significant circadian variability. Given that cortisol levels fluctuate heavily depending on the time of day, including this marker would have introduced substantial noise, potentially masking the specific stress-reactivity patterns we aimed to isolate. Fourth, potential confounders of lifestyle factors that may influence physiological and cytokine responses were not investigated. Finally, the cross-sectional design does not allow evaluation of whether this phenotype predicts clinically meaningful endpoints (e.g., future attacks, healthcare use, or longitudinal QoL trajectories).

### Strengths

This study also has several strengths. First, it adopts a multimodal approach, combining behavioral performance, subjective appraisal, cardiovascular indices, and inflammatory markers within the same experimental session, enabling convergent interpretation across domains. Second, it leverages a standardized and well-validated acute stress paradigm that provides an objective behavioral endpoint (withdrawal vs completion), which may be particularly informative in conditions characterized by different phenotypes and clinical heterogeneity such as in HAE-C1INH (13, 14). Third, the protocol includes repeated biological sampling, allowing the assessment of stress-related dynamics across the acute phase. Finally, by linking laboratory patterns to clinically meaningful patient-reported outcomes (AECT and AE-QoL), the study enhances translational relevance and supports the feasibility of using behavioral stress tolerance as a candidate marker in future longitudinal works.

## Conclusion

In this pilot study of HAE-C1INH, early withdrawal from the SECPT identified a subgroup with higher disease burden, greater subjective distress, attenuated pressor reactivity, and distinct inflammatory responses. While the exploratory nature of this research and the small sample size warrant caution in generalizing these results, the strength of our findings lies in the convergence across psychological, cardiovascular, and immunological domains. These preliminary findings support behavioral stress tolerance as a candidate psychobiological marker that may contribute to stratifying vulnerability in HAE-C1INH and guiding future longitudinal and mechanistic research. Preventive measures in addition to pharmacological prophylaxis may represent a more sustainable and synergistic approach that can contribute to a better management of patients affected by HAE-C1INH.

## Data Availability

The raw data supporting the conclusions of this article will be made available by the corresponding author upon reasonable request.

## References

[1] Wu MA Perego F Zanichelli A Cicardi M . Angioedema phenotypes: disease expression and classification. Clin Rev Allergy Immunol. (2016) 51:162–9. doi: 10.1007/s12016-016-8541-z, PMID: 27113957

[2] Maurer M Magerl M Betschel S Aberer W Ansotegui IJ Aygören-Pürsün E . The international WAO/EAACI guideline for the management of hereditary angioedema—The 2021 revision and update. Allergy. (2022) 77:1961–90. doi: 10.1111/all.15214, PMID: 35006617

[3] Longhurst H Bygum A . The humanistic, societal, and pharmaco-economic burden of angioedema. Clin Rev Allergy Immunol. (2016) 51:230–9. doi: 10.1007/s12016-016-8575-2, PMID: 27388236

[4] Chong-Neto HJ . A narrative review of recent literature of the quality of life in hereditary angioedema patients. World Allergy Organ J. (2023) 16:100758. doi: 10.1016/j.waojou.2023.100758, PMID: 36994443 PMC10040818

[5] Mendivil J Murphy R de la Cruz M Janssen E Boysen HB Jain G . Clinical characteristics and burden of illness in patients with hereditary angioedema: findings from a multinational patient survey. Orphanet J Rare Dis. (2021) 16:94. doi: 10.1186/s13023-021-01717-4, PMID: 33602292 PMC7893968

[6] Bork K Anderson JT Caballero T Craig T Johnston DT Li HH . Assessment and management of disease burden and quality of life in patients with hereditary angioedema: a consensus report. Allergy Asthma Clin Immunol. (2021) 17:40. doi: 10.1186/s13223-021-00537-2, PMID: 33875020 PMC8056543

[7] Banerji A . The burden of illness in patients with hereditary angioedema. Ann Allergy Asthma Immunol. (2013) 111:329–36. doi: 10.1016/j.anai.2013.08.019, PMID: 24125136

[8] Loli-Ausejo D López-Lera A Drouet C Lluncor M Phillips-Anglés E Pedrosa M . In search of an association between genotype and phenotype in hereditary angioedema due to C1-INH deficiency. Clin Rev Allergy Immunol. (2021) 61:1–14. doi: 10.1007/s12016-021-08834-9, PMID: 33469833

[9] Savarese L Mormile I Bova M Petraroli A Maiello A Spadaro G . Psychology and hereditary angioedema: A systematic review. Allergy Asthma Proc. (2021) 42:E1–7. doi: 10.2500/aap.2021.42.200073, PMID: 33404395 PMC7768073

[10] Erbay M Tuzer C Erkoç M Erkoç M . Earthquake as a trigger of acute attacks in people with hereditary angioedema. Dicle Tıp Dergisi. (2024) 51:46–53. doi: 10.5798/dicletip.1451503

[11] Eyice Karabacak D Demir S Yeğit OO Can A Terzioğlu K Ünal D . Impact of anxiety, stress and depression related to COVID-19 pandemic on the course of hereditary angioedema with C1-inhibitor deficiency. Allergy. (2021) 76:2535–43. doi: 10.1111/all.14796, PMID: 33650198 PMC8014132

[12] Christiansen SC Lopes Veronez C Smith TD Riedl MA Zuraw BL . Hereditary Angioedema: Impact of COVID-19 pandemic stress upon disease related morbidity and well-being. Allergy Asthma Proc. (2023) 44:115–21. doi: 10.2500/aap.2023.44.220096, PMID: 36872446 PMC9999437

[13] Schwabe L Haddad L Schachinger H . HPA axis activation by a socially evaluated cold-pressor test. Psychoneuroendocrinology. (2008) 33:890–5. doi: 10.1016/j.psyneuen.2008.03.001, PMID: 18403130

[14] Schwabe L Schächinger H . Ten years of research with the Socially Evaluated Cold Pressor Test: Data from the past and guidelines for the future. Psychoneuroendocrinology. (2018) 92:155–61. doi: 10.1016/j.psyneuen.2018.03.010, PMID: 29573884

[15] Whittaker AC Chauntry AJ . Blunted cardiovascular reactivity to acute psychological stress predicts low behavioral but not self-reported perseverance: A replication study. Psychophysiology. (2021) 58(1):e13707. doi: 10.1111/psyp.13707, PMID: 33068034

[16] De Maria B Ranucci L Gino C Cesoni Marcelli A Zingale LC Zulueta A . Functional physiological, psychological, and biochemical reactivity to socially evaluated cold pressor test in hereditary angioedema patients (FRoSEn). Front Immunol. (2026) 16:1736589. doi: 10.3389/fimmu.2025.1736589, PMID: 41573571 PMC12819172

[17] De Maria B Ranucci L Gino C Zulueta A Parati M Cesoni Marcelli A . Cardiac and vascular autonomic control in patients with hereditary angioedema. Front Physiol. (2025) 16:1690915. doi: 10.3389/fphys.2025.1690915, PMID: 41476578 PMC12747905

[18] Costantini M Musso M Viterbori P Bonci F Del Mastro L Garrone O . Detecting psychological distress in cancer patients: validity of the Italian version of the Hospital Anxiety and Depression Scale. Supportive Care Cancer. (1999) 7:121–7. doi: 10.1007/s005200050241, PMID: 10335929

[19] Mondo M Sechi C Cabras C . Psychometric evaluation of three versions of the Italian Perceived Stress Scale. Curr Psychol. (2021) 40:1884–92. doi: 10.1007/s12144-019-0132-8, PMID: 41746348

[20] Monticone M Baiardi P Ferrari S Foti C Mugnai R Pillastrini P . Development of the Italian version of the Pain Catastrophising Scale (PCS-I): cross-cultural adaptation, factor analysis, reliability, validity and sensitivity to change. Qual Life Res. (2012) 21:1045–50. doi: 10.1007/s11136-011-0007-4, PMID: 21912846

[21] Cerea S Todd J Ghisi M Mancin P Swami V . Psychometric properties of an Italian translation of the Functionality Appreciation Scale (FAS). Body Image. (2021) 38:210–8. doi: 10.1016/j.bodyim.2021.04.007, PMID: 33962221

[22] Casale S Prostamo A Giovannetti S Fioravanti G . Translation and validation of an Italian version of the Body Appreciation Scale-2. Body Image. (2021) 37:1–5. doi: 10.1016/j.bodyim.2021.01.005, PMID: 33545619

[23] Mehling WE Price C Daubenmier JJ Acree M Bartmess E Stewart A . The multidimensional assessment of interoceptive awareness (MAIA). PLoS One. (2012) 7:e48230. doi: 10.1371/journal.pone.0048230, PMID: 23133619 PMC3486814

[24] Sulla F Renati R Bonfiglio S Rollo D . “ Italian students and the Grit-S: A self-report questionnaire for measuring perseverance and passion for long-term goals.” In: 2018 IEEE international symposium on medical measurements and applications (MeMeA); 11 -13 June 2018; Rome, Italy. (2018). p. 1–5. doi: 10.1109/MeMeA.2018.8438668, PMID:

[25] Weller K Groffik A Magerl M Tohme N Martus P Krause K . Development and construct validation of the angioedema quality of life questionnaire. Allergy. (2012) 67:1289–98. doi: 10.1111/all.12007, PMID: 22913638

[26] Weller K Donoso T Magerl M Aygören-Pürsün E Staubach P Martinez-Saguer I . Validation of the angioedema control test (AECT)—A patient-reported outcome instrument for assessing angioedema control. J Allergy Clin Immunol Pract. (2020) 8:2050–2057.e4. doi: 10.1016/j.jaip.2020.02.038, PMID: 32173507

[27] Carroll D Ginty AT Whittaker AC Lovallo WR de Rooij SR . The behavioural, cognitive, and neural corollaries of blunted cardiovascular and cortisol reactions to acute psychological stress. Neurosci Biobehav Rev. (2017) 77:74–86. doi: 10.1016/j.neubiorev.2017.02.025, PMID: 28254428 PMC6741350

[28] Buzgoova K Balagova L Marko M Kapsdorfer D Riecansky I Jezova D . Higher perceived stress is associated with lower cortisol concentrations but higher salivary interleukin-1beta in socially evaluated cold pressor test. Stress. (2020) 23:248–55. doi: 10.1080/10253890.2019.1660872, PMID: 31466500

[29] Duschek S Werner NS Reyes del Paso GA . The behavioral impact of baroreflex function: A review. Psychophysiology. (2013) 50:1183–93. doi: 10.1111/psyp.12136, PMID: 24033333

[30] Bender L Weidmann H Rose-John S Renné T Long AT . Factor XII-driven inflammatory reactions with implications for anaphylaxis. Front Immunol. (2017) 8:1115. doi: 10.3389/fimmu.2017.01115, PMID: 28966616 PMC5605561

[31] Kaplan AP Joseph K . The bradykinin-forming cascade and its role in hereditary angioedema. Ann Allergy Asthma Immunol. (2010) 104:193–204. doi: 10.1016/j.anai.2010.01.007, PMID: 20377108

[32] Hornig B Kohler C Drexler H . Role of bradykinin in mediating vascular effects of angiotensin-converting enzyme inhibitors in humans. Circulation. (1997) 95:1115–8. doi: 10.1161/01.CIR.95.5.1115, PMID: 9054837

[33] De Maria B Parati M Bey Y Dalla Vecchia LA Perego F . Cardiovascular autonomic nervous system in a patient with hereditary angioedema affected by COVID-19. Cureus. (2024) 16(3):e56449. doi: 10.7759/cureus.56449, PMID: 38638792 PMC11025019

[34] Gramstad OR Schjalm C Mollnes TE Nielsen EW . Increased thromboinflammatory load in hereditary angioedema. Clin Exp Immunol. (2023) 214:170–81. doi: 10.1093/cei/uxad091, PMID: 37561062 PMC10714191

[35] Tracey KJ . The inflammatory reflex. Nature. (2002) 420:853–9. doi: 10.1038/nature01321, PMID: 12490958

[36] McEwen BS . Physiology and neurobiology of stress and adaptation: central role of the brain. Physiol Rev. (2007) 87:873–904. doi: 10.1152/physrev.00041.2006, PMID: 17615391

[37] Barrett LF Simmons WK . Interoceptive predictions in the brain. Nat Rev Neurosci. (2015) 16:419–29. doi: 10.1038/nrn3950, PMID: 26016744 PMC4731102

[38] Treadway MT Cooper JA Miller AH . Can’t or won’t? Immunometabolic constraints on dopaminergic drive. Trends Cognit Sci. (2019) 23:435–48. doi: 10.1016/j.tics.2019.03.003, PMID: 30948204 PMC6839942

[39] Noakes TD . The central governor model of exercise regulation applied to the marathon. Sports Med. (2007) 37:374–7. doi: 10.2165/00007256-200737040-00026, PMID: 17465612

[40] Theoharides TC Alysandratos K-D Angelidou A Delivanis D-A Sismanopoulos N Zhang B . Mast cells and inflammation. Biochim Biophys Acta (BBA) - Mol Basis Dis. (2012) 1822:21–33. doi: 10.1016/j.bbadis.2010.12.014, PMID: 21185371 PMC3318920

[41] Nguyen SMT Rupprecht CP Haque A Pattanaik D Yusin J Krishnaswamy G . Mechanisms governing anaphylaxis: inflammatory cells, mediators, endothelial gap junctions and beyond. Int J Mol Sci. (2021) 22:7785. doi: 10.3390/ijms22157785, PMID: 34360549 PMC8346007

