# Supplementary material for: Behavioural withdrawal during an acute stress test as a marker of psychobiological vulnerability in hereditary angioedema
Source: Front Immunol. 2026 Mar 3;17:1784326. doi: 10.3389/fimmu.2026.1784326 (PMC12991979; doi:10.3389/fimmu.2026.1784326)
Supplement: Supplementary file 1 [file Table1.docx]

**APPENDIX A**

**APPENDIX A.** Mean cardiovascular differences (Δ) across experimental phases and between groups comparison (Completers vs Non-completers).

| **CVR INDICES** | **GROUPS** | **∆REST-SECPT** | **∆REST-POST10** | **∆REST-POST40** | **∆SECPT-POST10** | **∆SECPT-POST40** | **∆POST10-POST40** |
| --- | --- | --- | --- | --- | --- | --- | --- |
| **HR, bpm** | Completers | 7.1 ± 5.8 | -0.8 ± 4.2 | -0.7 ± 6.1 | -7.9 ± 5.2 | -7.9 ± 6.1 | 0.06 ± 4.5 |
|  | Non-completers | 8.1 ± 3.8 | -1.9 ± 3.9 | -2.6 ± 5.3 | -10.0 ± 5.9 | -10.8 ± 6.4 | -0.7 ± 1.9 |
| **SAP, mmHg** | Completers | 26.2 ± 12.0 | -0.03 ± 17.0 | 7.5 ± 14.8 | -26.2 ± 18.2 | -18.2 ± 11.6 | 5.0 ± 12.5 |
|  | Non-completers | 9.1 ± 7.9***** | 0.5 ± 5.3 | -6.0 ± 13.2 | -8.5 ± 7.9 | -15.2 ± 18.1 | -6.5 ± 11.9 |
| **DAP, mmHg** | Completers | 14.5 ± 5.2 | 0.3 ± 8.1 | 5.8 ± 10.7 | -14.2 ± 7.9 | -9.0 ± 9.7 | 3.8 ± 10.4 |
|  | Non-completers | 6.4 ± 4.5***** | 3.0 ± 1.3 | 2.8 ± 8.2 | -3.4 ± 4.0***** | -3.6 ± 8.8 | -0.2 ± 7.9 |

**Note**. CVR: Cardio Vascular Reactivity; REST: 10 minutes resting phase; SECPT: Socially Evaluated Cold Pressor Test; POST10: 10 minutes after SECPT; POST40: 30 minutes after POST10; HR: heart rate; SAP: systolic arterial pressure; DAP: diastolic arterial pressure; Completers: participants who completed the SECPT; Non-completers: participants who did not complete the SECPT. ∆: differences between phases. Data are presented as mean ± Standard deviation. *Completers vs Non-completers, *p*≤0.05
