# Supplementary material for: Behavioural withdrawal during an acute stress test as a marker of psychobiological vulnerability in hereditary angioedema
Source: Front Immunol. 2026 Mar 3;17:1784326. doi: 10.3389/fimmu.2026.1784326 (PMC12991979; doi:10.3389/fimmu.2026.1784326)
Supplement: Supplementary file 2 [file Table2.docx]

**APPENDIX B.** Mean concentration differences (Δ) of inflammatory markers across experimental phases and between groups comparison (Completers vs Non-completers).

| **CYTOKINE** | **GROUP** | **∆REST-POST10** | **∆REST-POST40** | **∆POST10-POST40** |
| --- | --- | --- | --- | --- |
| **IL-1β, pg/ml** | Completers | -0.04 ± 1.29 | 0.19 ± 1.75 | 0.23 ± 1.01 |
|  | Non-completers | -1.42 ± 4.81 | -1.36 ± 2.34 | 0.06 ± 2.56 |
| **TNF-α, pg/ml** | Completers | -0.20 ± 0.90 | -0.46 ± 0.86 | -0.26 ± 0.76 |
|  | Non-completers | 0.21 ± 0.99 | 0.93 ±0.97* | 0.72 ± 0.75* |
| **IL-6, pg/ml** | Completers | 0.16 ± 0.92 | -0.15 ± 0.76 | -0.31 ± 0.86 |
|  | Non-completers | 0.12 ± 1.95 | 0.12 ± 1.87 | -0.01 ± 0.83 |

**Note**. REST:10 minutes resting phase; POST10: 10 minutes after Socially Evaluated Cold Pressor Test; POST40: 30 minutes after POST10; IL-1β: Interleukin-1 beta; TNF-α: Tumor Necrosis Factor-alpha; IL-6: Interleukin-6; Completers: participants who completed the SECPT; Non-completers: participants who did not complete the SECPT. ∆: differences between phases. Data are presented as mean ± Standard deviation. *Completers vs Non-completers, *p*≤0.05
